# Supplementary material for: Silicone Breast Implant Coated with Triamcinolone Inhibited Breast-Implant-Induced Fibrosis in a Porcine Model
Source: Materials (Basel). 2021 Jul 14;14(14):3917. doi: 10.3390/ma14143917 (PMC8307199; doi:10.3390/ma14143917)
Supplement: Supplementary file 1 [file materials-14-03917-s001.zip › materials-1273438-supplementary.pdf]

materials

MDPI

Supporting information

Silicone Breast Implant Coated with Triamcinolone Inhibited Breast-Implant-Induced Fibrosis in a Porcine Model

Sun-Young Nam [1,†], Han Bi Ji [2,†], Byung Ho Shin [1], Pham Ngoc Chien [1], Nilsu Donmez [1], Xin Rui Zhang [1,3], Beom Kang Huh [2], Min Ji Kim [2], Young Bin Choy [2,4,5,*] and Chan Yeong Heo [1,3,*]

1 Department of Plastic and Reconstructive Surgery, Seoul National University Bundang Hospital, Seongnam 13620, Korea; 99261@snubh.org (S.-Y.N.); shinzmatt@naver.com (B.H.S.); ngocchien1781@gmail.com (P.N.C.); nlsdonmez@gmail.com (N.D.); zhangxinrui@snu.ac.kr (X.R.Z.)
2 Interdisciplinary Program in Bioengineering, College of Engineering, Seoul National University, Seoul 08826, Korea; hanbi2697@snu.ac.kr (H.B.J.); bkhuh85@snu.ac.kr (B.K.H.); kmj346@snu.ac.kr (M.J.K.)
3 Department of Plastic and Reconstructive Surgery, College of Medicine, Seoul National University, Seoul 03080, Korea
4 Institute of Medical & Biological Engineering, Medical Research Center, Seoul National University, Seoul 03080, Korea
5 Department of Biomedical Engineering, Seoul National University College of Medicine, Seoul 03080, Korea
* Correspondence: ybchoy@snu.ac.kr (Y.B.C.); lionheo@snu.ac.kr (C.Y.H.)
† The authors contributed equally to this work.

Materials & Methods

Western blot analysis of specific markers related to CC

For an insight into the factors closely associated with the formation of capsules following the insertion of silicone into the tissue, Western Protein expressions blot analysis was performed for specific fibrosis markers like α-SMA and TGFβ1. The protocol for Western blot was followed as described earlier by Park et al. [1] The α-SMA and TGFβ1 protein expression levels were observed in capsular tissue in the Image J measurements tool quantified with total grey values for each band and then normalized with the respective β-actin. All the experiments were performed in triplicates and the mean values were noted.

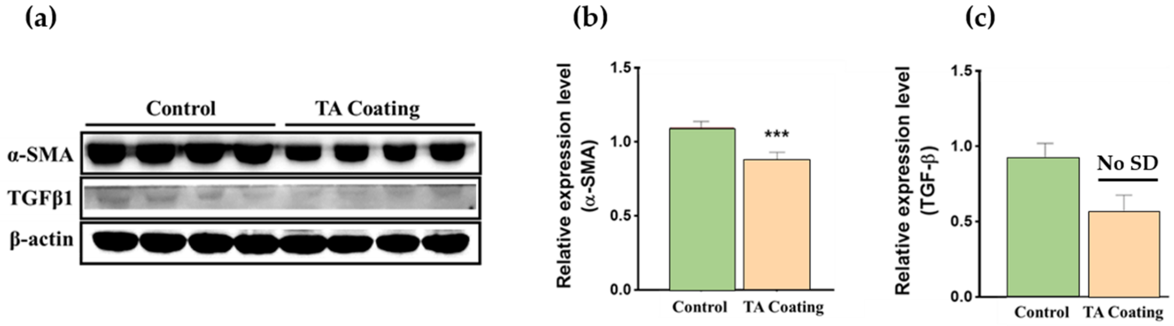

Figure S1. Expression of fibrosis marker proteins. (**a**) Protein expression pattern of α-SMA and TGFβ1 in control and TA coated silicone implant groups; (**b**) relative expression of α-SMA and (**c**) TGFβ1 with respect to β-actin was represented, asterisks *** and "No SD" indicate the significant difference ($p < 0.001$) and no significant difference, respectively. Data represented as mean ± SEM.

References

1. Park, C.; Lee, S.-W.; Kim, J.; Song, E.-H.; Jung, H.-D.; Park, J.-U.; Kim, H.-E.; Kim, S. and Jang, T.-S. Reduced fibrous capsule formation at nano-engineered silicone surfaces via tantalum ion implantation. *Biomater Sci*. **2019**, *7*, 2907-2919, doi: 10.1039/C9BM00427K.
